# Adsorption of Helium and Hydrogen on Triphenylene and 1,3,5-Triphenylbenzene

**DOI:** 10.3390/molecules27154937

**Published:** 2022-08-03

**Authors:** Stefan Bergmeister, Siegfried Kollotzek, Florent Calvo, Elisabeth Gruber, Fabio Zappa, Paul Scheier, Olof Echt

**Affiliations:** 1 Institut für Ionenphysik und Angewandte Physik, Universität Innsbruck, A-6020 Innsbruck, Austria; stefan.bergmeister@uibk.ac.at (S.B.); siegfried.kollotzek@uibk.ac.at (S.K.); e.gruber@uibk.ac.at (E.G.); fabio.zappa@uibk.ac.at (F.Z.); paul.scheier@uibk.ac.at (P.S.); 2 Laboratoire Interdisciplinaire de Physique, CNRS, Université Grenoble Alpes, F-38000 Grenoble, France; 3 Department of Physics, University of New Hampshire, Durham, NH 03824, USA

**Keywords:** noble gases, helium, hydrogen, PAH, triphenylene, 1,3,5-triphenylbenzene, adsorption, mass spectrometry, path-integral molecular dynamics simulations

## Abstract

The adsorption of helium or hydrogen on cationic triphenylene (TPL, C_18_H_12_), a planar polycyclic aromatic hydrocarbon (PAH) molecule, and of helium on cationic 1,3,5-triphenylbenzene (TPB, C_24_H_18_), a propeller-shaped PAH, is studied by a combination of high-resolution mass spectrometry and classical and quantum computational methods. Mass spectra indicate that He*_n_*TPL^+^ complexes are particularly stable if *n* = 2 or 6, in good agreement with the quantum calculations that show that for these sizes, the helium atoms are strongly localized on either side of the central carbon ring for *n* = 2 and on either side of the three outer rings for *n* = 6. Theory suggests that He_14_TPL^+^ is also particularly stable, with the helium atoms strongly localized on either side of the central and outer rings plus the vacancies between the outer rings. For He*_n_*TPB^+^, the mass spectra hint at enhanced stability for *n* = 2, 4 and, possibly, 11. Here, the agreement with theory is less satisfactory, probably because TPB^+^ is a highly fluxional molecule. In the global energy minimum, the phenyl groups are rotated in the same direction, but when the zero-point harmonic correction is included, a structure with one phenyl group being rotated opposite to the other two becomes lower in energy. The energy barrier between the two isomers is very small, and TPB^+^ could be in a mixture of symmetric and antisymmetric states, or possibly even vibrationally delocalized.

## 1. Introduction

When inert gas atoms are physisorbed on a cold, highly structured carbonaceous surface such as that of graphite, a nanotube, fullerene, or coronene molecule, it is tempting to expect that the corrugation of the substrate will favor the formation of a commensurate adsorption layer. After all, a single inert gas atom will preferentially adsorb in the hollow of a carbon ring. He_32_C_60_^+^ has, indeed, an evaporation energy that significantly exceeds that of He_33_C_60_^+^ because its 20 hexagonal and 12 pentagonal carbon rings are decorated by one helium atom each [1,2,3]. The commensurate layer on C_60_^+^ is energetically favored even though its first solvation layer can accommodate several additional helium atoms [1,3,4,5].

The formation of a commensurate layer on C_60_^+^ is also favored for the adsorption of non-polar molecules including H_2_, N_2_, O_2_, CH_4_, and C_2_H_4_ [6,7,8,9,10,11,12], or for He*_n_*C_60_^−^ anions [13]. In contrast, the non-polar CO_2_ molecules do not form a commensurate layer on charged C_60_, and its quadrupole moment renders its adsorption sensitive to the charge state of the substrate [14].

However, several factors may affect the formation of an adsorbate layer that is in registry with the substrate. One such factor is the size of the adsorbate atoms or molecules relative to the distance between favored adsorption sites. For graphene or planar graphitic surfaces, the distance between preferred adsorption sites is only 0.246 nm, less than the nearest neighbor distance in any van der Waals bound condensed system, including helium and hydrogen, thus preventing the formation of a commensurate 1 × 1 layer. A way out of this predicament is the formation of the √3 × √3 phase, observed for He, H_2_, and many molecular adsorbates, in which all second nearest hexagonal carbon rings are occupied [15].

Another important factor is the strength of the attractive interaction between the adsorbate and the substrate relative to that between adsorbate species, and the possible existence of anisotropic interaction terms in the latter. According to a theoretical study, xenon is the only heavy noble gas that favors the formation of a commensurate layer on C_60_^+^ [16], and mass spectra do not reveal any preference for the adsorption of 32 water or ammonia molecules on C_60_^+^ [9,17].

Quantum effects may also hinder the formation of a commensurate adsorption layer. Helium atoms and hydrogen molecules adsorbed on fullerenes [1,2,3,4,12] or polycyclic aromatic hydrocarbons (PAHs) [18,19,20,21,22,23,24,25,26,27,28,29] may exhibit strong vibrational delocalization, depending on the density of the adsorbed layer and the charge of the substrate.

In our recent published work, we combined experimental techniques (high-resolution mass spectrometry and optical absorption spectroscopy) and path-integral molecular dynamics simulations to study the adsorption of He and H_2_ on cationic planar PAHs, including anthracene (C_14_H_10_), phenanthrene (C_14_H_10_), fluoranthene (C_16_H_10_), pyrene (C_16_H_10_), and coronene (C_24_H_12_) [26,29,30,31,32]. One might surmise that in these systems, the crowded 1 × 1 phase is stabilized relative to adsorption on extended graphitic surfaces because the helium atoms adsorbed on the outer rings can relax outwards. However, neither experiment nor theory support this naïve view, partly because quantum effects will destabilize the system, and partly because the peripheral HCCH-bonds offer additional favorable sites [24,26,33]. We also studied hexaphenylbenzene (HPB, C_42_H_30_), a propeller-shaped molecule. Here, one finds the enhanced stability of a phase akin to the 1 × 1 phase, thanks to the increased spatial separation between the carbon rings. However, the facile out-of-plane rotation of the phenyl groups leads to several nearly isoenergetic isomers for which the presence of helium atoms is likely to affect the tilt of the phenyl groups, and treating HPB^+^ as a rigid substrate may be too simplistic.

In the present paper, we explore the adsorption of He and H_2_ on the rigid, planar cationic triphenylene (TPL, C_18_H_12_), and of helium on the highly fluxional, cationic 1,3,5-triphenylbenzene (TPB, C_24_H_18_). Mass spectra are recorded by first forming helium nanodroplets (HNDs) in a supersonic expansion, ionizing the droplets by collisions with energetic electrons, passing the mass-to-charge selected HNDs through dilute TPL or TPB vapor leading to the capture of molecules in the droplets, gently stripping excess helium from the ions in an evaporation cell filled with helium gas at room temperature, and analyzing the size distribution of the emerging He*_n_*TPL^+^ or He*_n_*TPB^+^ ions in a high-resolution time-of-flight mass spectrometer. (H_2_)*_n_*TPL^+^ ions are formed by introducing H_2_ into the evaporation cell. Local anomalies in the ion abundance versus size *n* suggest the enhanced stability of certain sizes.

To assist with the interpretation of these measurements, we also carried out an atomistic modeling of He*_n_*TPL^+^, (H_2_)*_n_*TPL^+^, and He*_n_*TPB^+^ using the same computational methodology as in earlier works [2,28,29,33]. In TPB^+^ the phenyl groups are rotated out of the plane and two nearly isoenergetic isomers exist, one with all blades tilted in the same direction, and another with one blade tilted opposite to the other two. Global optimization by basin-hopping was also performed to identify low-energy structural candidates for 1 to 50 He atoms or H_2_ molecules attached to the hydrocarbon ion, keeping its geometry fixed.

## 2. Results and Discussions

### 2.1. Helium and Hydrogen Adsorbed on Triphenylene (TPL) Cations

**Figure 1:**
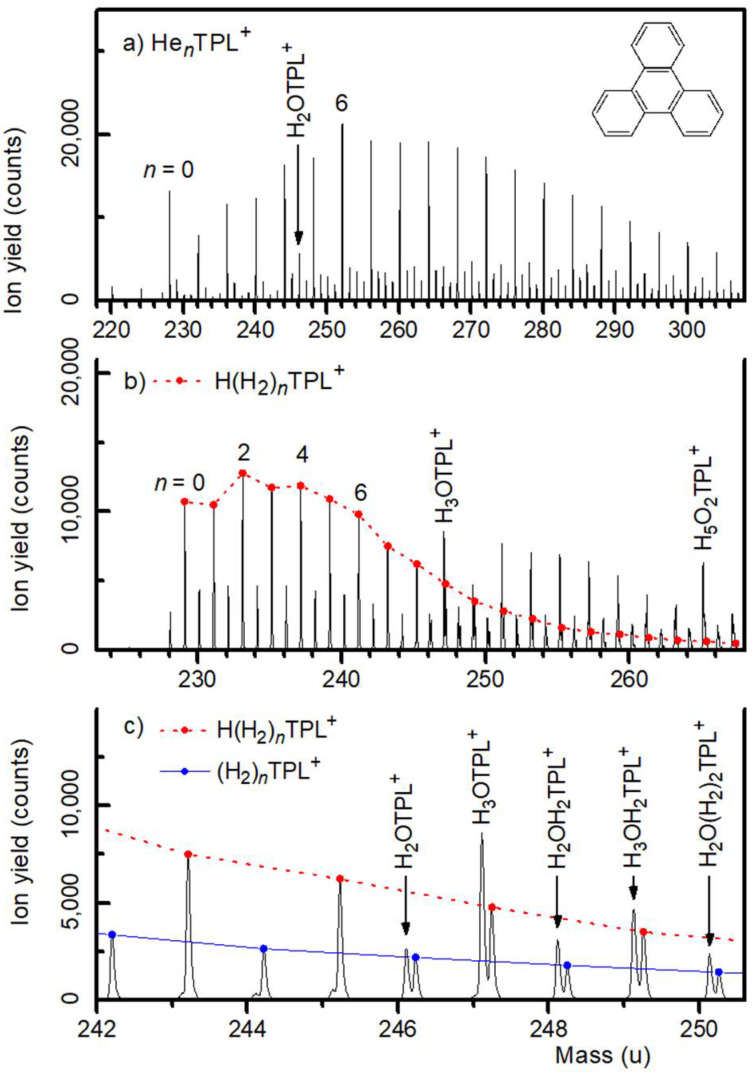
Panels (**a**,**b**): Mass spectra of triphenylene (TPL, C_18_H_12_, mass 228.094 u) cations complexed with helium and hydrogen, respectively. In panel (**b**), mass peaks due to protonated H(H_2_)*_n_*TPL^+^ ions are connected by a dotted line. Panel (**c**) shows a narrow section of the spectrum in panel (**b**). Mass peaks due to H(H_2_)*_n_*TPL^+^ and (H_2_)*_n_*TPL^+^ are connected by dotted and solid lines, respectively. Mass peaks arising from a water contamination are resolved.

A mass spectrum of HNDs doped with triphenylene (TPL, C_18_H_12_, CAS Registry Number 217-59-4) is displayed in Figure 1a. The pressure in the evaporation cell was set to *P*_He_ = 0.087 Pa (corrected for the sensitivity of the ion gauge). The series of prominent mass peaks, commencing at 228 u and spaced at 4 u, is due to He*_n_*^12^C_18_H_12_^+^, *n* ≥ 0. Isotopologues of He*_n_*C_18_H_12_^+^ that contain 1, 2, or 3 atoms of ^13^C (natural abundance 1.07%) give rise to weaker satellite peaks. Mass peaks at 220 and 224 u are due to bare He_55_^+^ and He_56_^+^, respectively.

The mass peak at 246 u is due to H_2_O^12^C_18_H_12_^+^; this ion complexed with helium gives rise to another series of mass peaks midway between the isotopically pure He*_n_*TPL^+^ peaks. Ions containing one H_2_O cause no problem in the data analysis but the mass of two H_2_O is, within 0.002 u, the same as that of nine He atoms; therefore we cannot resolve (H_2_O)_2_He*_n_*TPL^+^ and He*_n_*_+9_TPL^+^ mass peaks.

Doping HNDs with TPL and molecular hydrogen results in protonated H(H_2_)*_n_*TPL^+^ ions and a weaker series of unprotonated (H_2_)*_n_*TPL^+^ ions, see Figure 1b. A water contamination gives rise to two other ion series, namely H_3_O(H_2_)*_n_*TPL^+^ and H_2_O(H_2_)*_n_*TPL^+^. The nominal mass of these ions coincides with that of H(H_2_)*_n_*_+9_TPL^+^ and (H_2_)*_n_*_+9_TPL^+^, respectively. However, the mass of (H_2_)_9_ exceeds that of H_2_O by 0.130 u, and the corresponding mass peaks are clearly resolved as seen in the expanded mass spectrum in Figure 1c.

**Figure 2:**
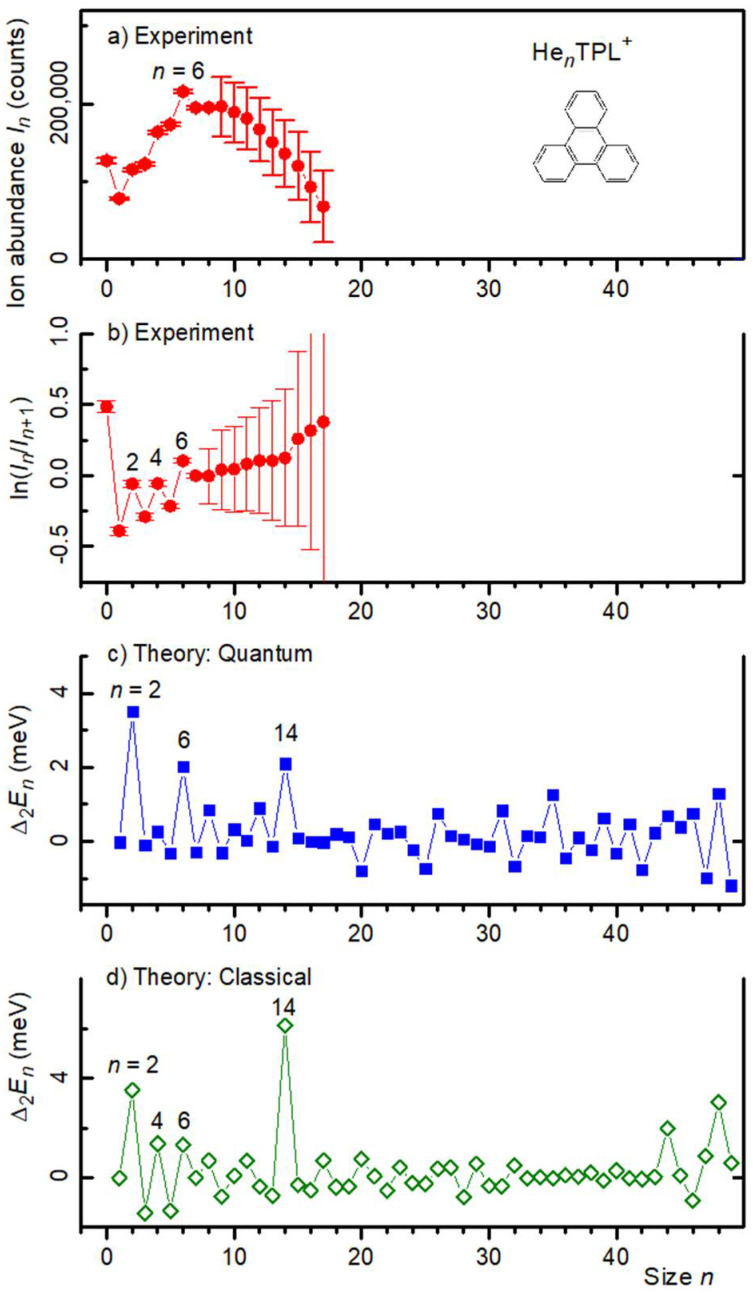
Panel (**a**): Ion abundance *I_n_* of He*_n_*TPL^+^ versus size *n*. Panel (**b**): The first derivative of the logarithm of *I_n_*. Prominent local anomalies are labeled. Panel (**c**): The second derivative of the energy *E_n_* of He*_n_*TPL^+^ calculated from the quantum virial energy. Panel (**d**): Same as panel (**c**) but calculated for the classical global minima.

The envelope of the ion series He*_n_*TPL^+^ in Figure 1a is smooth except for a slight enhancement of the He_6_TPL^+^ mass peak. This and other local anomalies can be identified more reliably when the mass spectrum is processed with the software IsotopeFit, which accounts for isotopic patterns and assigns appropriate contributions of ions that have the same nominal mass [34]. The resulting ion abundance *I_n_* is plotted versus size *n* in Figure 2a. Note that the error bar of *I_n_* increases abruptly above *n* = 8 because of the above-mentioned mass coincidence of He_9_ and 2 H_2_O.

In order to reveal local anomalies in the ion abundance more clearly, we plotted the negative first derivative (−∆_1_) of the logarithmic ion abundance in Figure 2b. This quantity equals approximately the 2nd derivative (Δ_2_) of the Helmholtz free energy *F_n_* divided by the Boltzmann constant *k*_B_ and vibrational temperature *T*,

(1) $$-\Delta_{1}lnI_{n}=ln\frac{I_{n}}{I_{n+1}}≅\Delta_{2}\frac{F_{n}}{k_{B}T}$$

provided that the observed cluster ions are the evaporative products of larger clusters [35,36]. This condition was clearly met in our experiments. The concept of cluster temperature is subtle, and anomalies in the binding energy *E_n_* will be accompanied by anomalies in *T* [37,38]. However, if one ignores anomalies in the temperature and in the size dependence of the entropy, one expects

(2) $$-\Delta_{1}lnI_{n}≅const\;\Delta_{2}E_{n}$$

The graph of −∆_1_*lnI_n_* in Figure 2b reveals statistically significant local maxima at *n* = 2, 4, and 6, suggesting that for these sizes, the 2nd derivative of the energy *E_n_*, or the 1st derivative of the dissociation (or evaporation) energy *D_n_* = −∆_1_*E_n_*_,_ is enhanced. In other words, one expects that TPL^+^ complexed with *n* = 2, 4, or 6 helium atoms enjoys enhanced stability.

A comparison with atomistic calculations is afforded by the data in Figure 2c, which show the second derivatives of the virial energies *E_n_*. For completeness, we also show (in Figure 2d) the 2nd derivatives of the classical energies (without zero-point correction), although these data are not likely to correctly describe the properties of He adsorbed on PAHs; they will not be discussed further but are provided as they might serve for future reference. The anomalies in the experimental data at *n* = 2 and 6 are reproduced in the quantum calculations, but the anomaly at *n* = 4 is not as clear from the quantum data. A pronounced anomaly in the quantum energies at *n* = 14 is not mirrored in the experimental data, but the large experimental error bars in this size range may possibly hide the anomaly.

**Figure 3:**
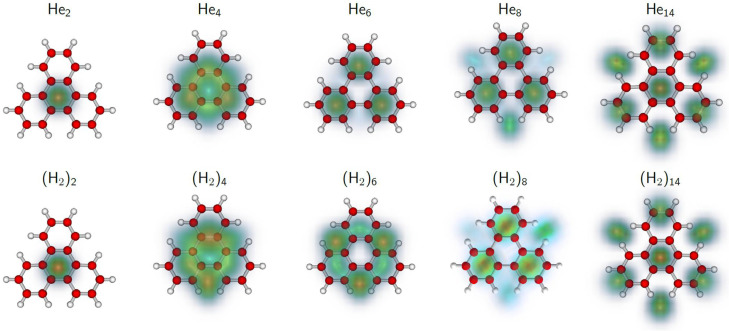
Selected structures of He*_n_*TPL^+^ and (H_2_)*_n_*TPL^+^ clusters with *n* = 2, 4, 6, 8, and 14. For each size, the helium and H_2_ densities obtained from the PIMD simulations are superimposed on the structure of TPL^+^.

Helium atoms bound to graphitic substrates or aromatic molecules tend to be most strongly bound atop the carbon rings [1,26,29,30,33]. Cluster sizes of enhanced stability often correlate with an arrangement of the adsorbate layer that is commensurate with the substrate. Computed structures of TPL^+^ with *n* = 2, 4, 6, 8, and 14 He atoms attached are displayed in the upper row of Figure 3; structures for *n* = 10, 12, 16, 18, 30 are provided as Supplementary Material (Figure S6). For each size, the helium densities obtained from the PIMD simulations are superimposed on the structure of TPL^+^. For *n* = 2, 6, and 14, the helium atoms are strongly localized: For *n* = 2, the He atoms are adsorbed on either side of the central carbon ring; for *n* = 6, they are adsorbed on either side of the three outer rings; and for *n* = 14, they are adsorbed on either side of the central and outer rings plus the vacancies between the outer rings. Note that for *n* = 2, 6, and 14, the adsorbate layer and the substrate exhibit the same three-fold rotational symmetry. Furthermore, for these sizes, the systems are particularly stable (Figure 2c).

For sizes other than *n* = 2, 6, 14, the helium atoms are not well localized. For *n =* 4, the preferred quantum structure has 2 helium atoms on either side of the hydrocarbon cation (a 2 + 2 configuration), whereas the classical global minimum is found for the 3 + 1 configuration. However, the difference in quantum energies between the 3 + 1 and 2 + 2 configurations is very small (0.22 meV), hence, it is also possible that both configurations coexist in the experiment, thereby possibly explaining a part of the observed disagreement. For *n =* 8, the three outer carbon rings are occupied on either side, the remaining two helium atoms being delocalized on the periphery. Such a growth pattern eventually leads to the structure predicted for *n =* 14, in which the 7 + 7 arrangement is particularly stable. However, because the size of helium (≈0.3 nm) exceeds the distance between the centers of adjacent carbon rings in a graphitic lattice (0.246 nm), the He atoms adsorbed at the outer ring are pushed to the periphery. The arrangement in complexes containing 16 or 18 He atoms (see Figure S6) shows the appearance of a halo that surrounds the highly ordered He_14_TPL^+^. For *n* = 30, though, the He atoms in the second solvation shell show some degree of localization.

**Figure 4:**
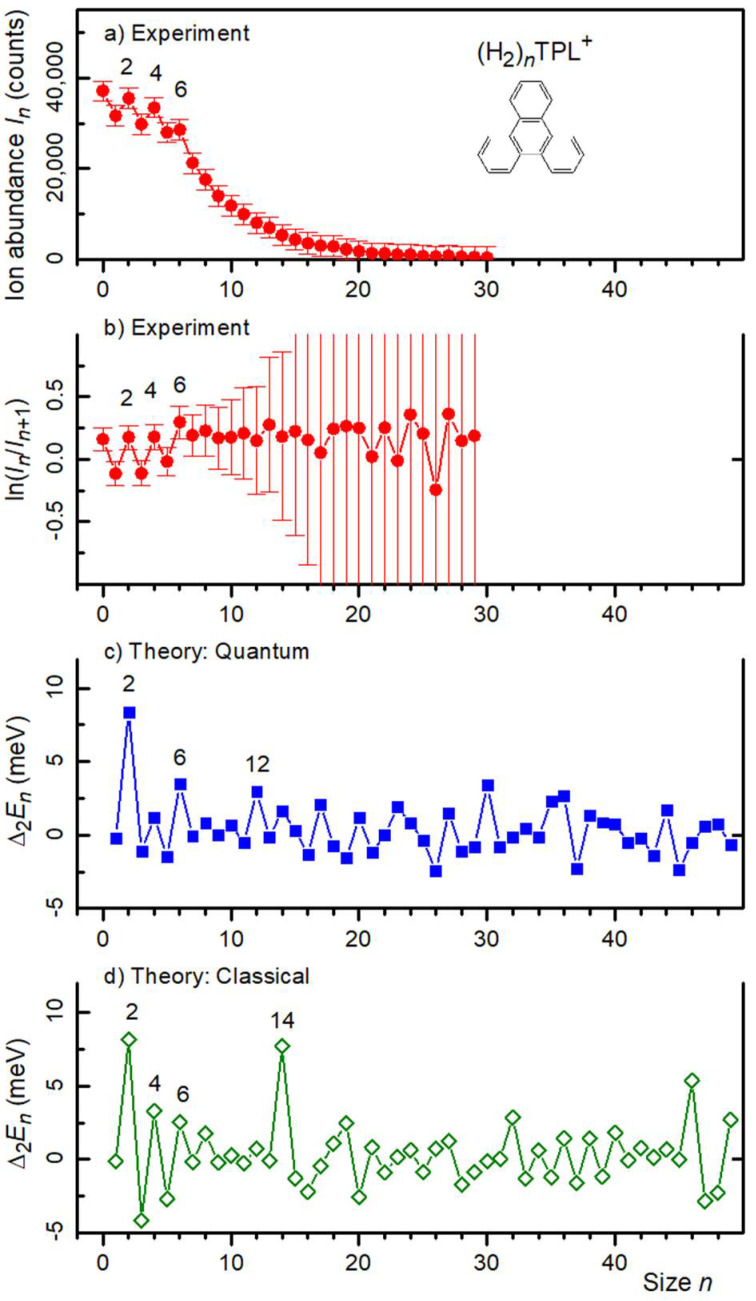
Panel (**a**): Ion abundance *I_n_* of (H_2_)*_n_*TPL^+^ versus size *n*. Panel (**b**): The first derivative of the logarithm of *I_n_*. Prominent local anomalies are labeled. Panel (**c**): The second derivative of the energy *E_n_* of (H_2_)*_n_*TPL^+^ calculated from the quantum virial energy. Panel (**d**): Same as panel (**c**) but calculated for the classical global minima.

Figure 4a,b display the ion abundance and its first logarithmic derivative, respectively, of (H_2_)*_n_*TPL^+^. Local anomalies at *n* = 2, 4, and 6 are clearly visible. These values agree with the anomalies observed for He*_n_*TPL^+^.

*I_n_* and −∆_1_*lnI_n_* for H(H_2_)TPL^+^ and H_3_O(H_2_)*_n_*TPL^+^ are provided as Supplementary Material (Figures S1 and S2, respectively). The only statistically significant anomaly in these data is a local maximum at *n* = 2.

The 2nd derivatives of the computed energies of (H_2_)*_n_*TPL^+^ are shown in Figure 4c,d for the quantum and classical approach, respectively. The quantum data reveal anomalies at 2 and 6, just as for He*_n_*TPL^+^, but the anomaly predicted for the He*_n_*TPL^+^ energies at *n* = 14 is absent. Instead one observes a minor anomaly at *n* = 12. For some values of *n*, the computed structures of (H_2_)*_n_*TPL^+^ (Figure 3 and Figure S5) closely resemble those of He*_n_*TPL^+^ while there are significant differences for other sizes. For *n* = 4, the 3 + 1 pattern is now preferred (by about 1 meV) over 2 + 2 in the quantum and classical calculations. For *n* = 6, 10, and 12 the differences between H_2_ and He adsorption patterns are particularly striking.

### 2.2. Helium Adsorbed on 1,3,5-Triphenylbenzene (TPB) Cations

Mass spectra of HNDs doped with 1,3,5-triphenylbenzene (TPB, C_24_H_18_, CAS Registry Number 612-71-5) are qualitatively similar to the spectrum displayed in Figure 1 for HNDs doped with TPL, showing a prominent series of mass peaks due to isotopically pure He*_n_*TPB^+^, weaker satellite series due to isotopologues of TPB, ions containing a water impurity, and bare He*_n_*^+^. Sample mass spectra are provided as Supplementary Material (Figure S3).

**Figure 5:**
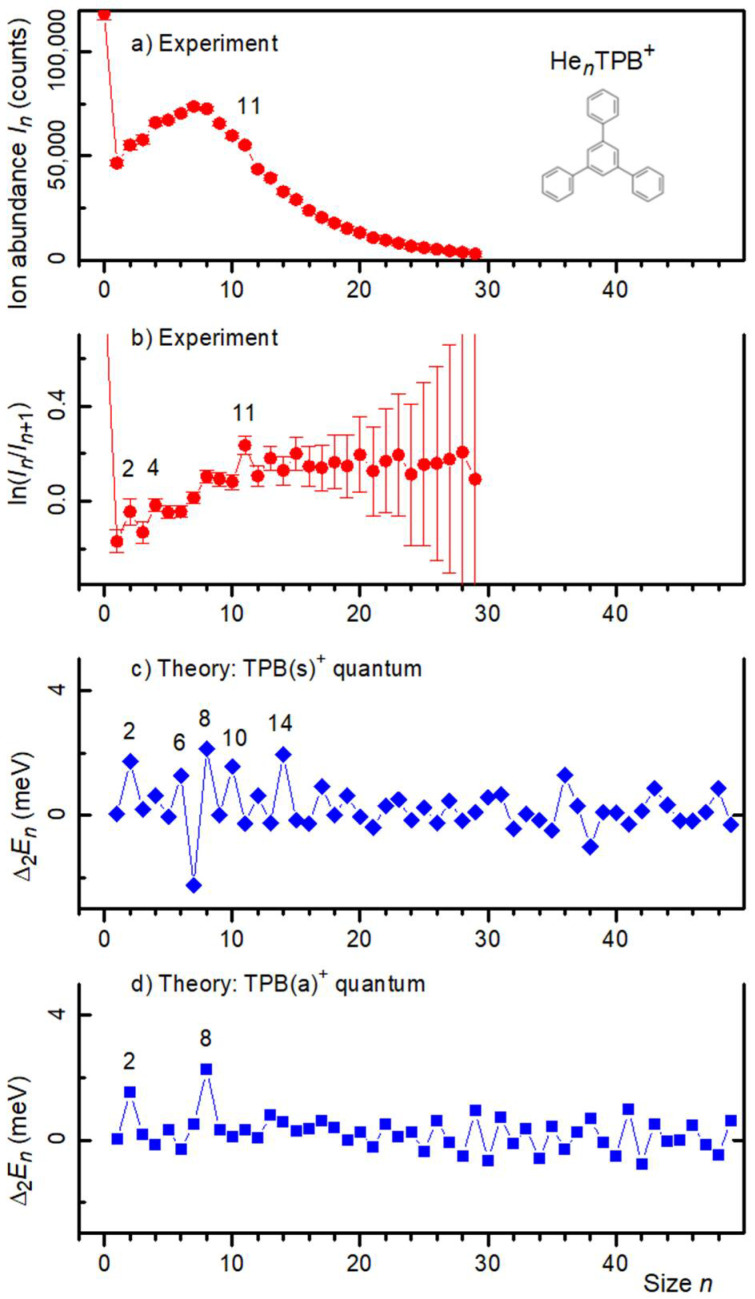
Panel (**a**): Ion abundance *I_n_* of He*_n_*TPB^+^ versus size *n*. Panel (**b**): The first derivative of the logarithm of *I_n_*. Prominent local anomalies are labeled. Panels (**c**,**d**): The second derivative of the energy *E_n_* of He*_n_*TPB^+^ calculated from the quantum virial energy for the symmetric (s) and asymmetric (a) isomer, respectively.

The ion abundance of He*_n_*TPB^+^ and its first logarithmic derivative are plotted in Figure 5a,b, respectively. The pressure in the evaporation cell was set to *P*_He_ = 0.089 Pa. The data in panel b suggest enhanced stability of ions containing *n* = 2, 4, or 11 helium atoms.

However, the above-mentioned mass coincidence between He_9_ and 2 H_2_O corrodes the reliability of the ion abundance for *n* ≥ 9, and of the 1st logarithmic derivative for *n* ≥ 8. We cannot decrease the H_2_O contamination to an amount that may be safely neglected, but we can explore how a deliberate increase in the water contamination affects the data. This has been achieved by increasing *P*_He_ in the evaporation cell (residual water in that cell is the main source of the water contamination). Three representative mass spectra are displayed in Figure S3. The spectrum in Figure S3a was recorded with the smallest value of *P*_He_; it provides the data for Figure 5a,b. As *P*_He_ is increased, the yield of the well-resolved H_2_OTPB^+^ mass peak increases relative to the adjacent strong mass peaks that are due to He_4_TPL^+^ and He_5_TPL^+^. We thus expect that the relative yield of the unresolved (H_2_O)_2_TPB^+^ mass peak will also increase as *P*_He_ is increased and, indeed, a pronounced anomaly at nominally *n* = 9 emerges in the first logarithmic derivative (Figure S3f). This anomaly is clearly due to (H_2_O)_2_TPB^+^ rather than He_9_TPB^+^.

At the lowest value of *P*_He_, i.e., at the lowest degree of water contamination, we observe a small enhancement of the first derivative at *n* = 8 (see Figure 5b). It is tempting to assume that the enhancement at *n* = 8 would be even stronger if we could completely eliminate the H_2_O contamination. In other words, the experimental data possibly indicate that He_8_TPB^+^ enjoys enhanced stability, but we cannot tell for sure.

**Figure 6:**
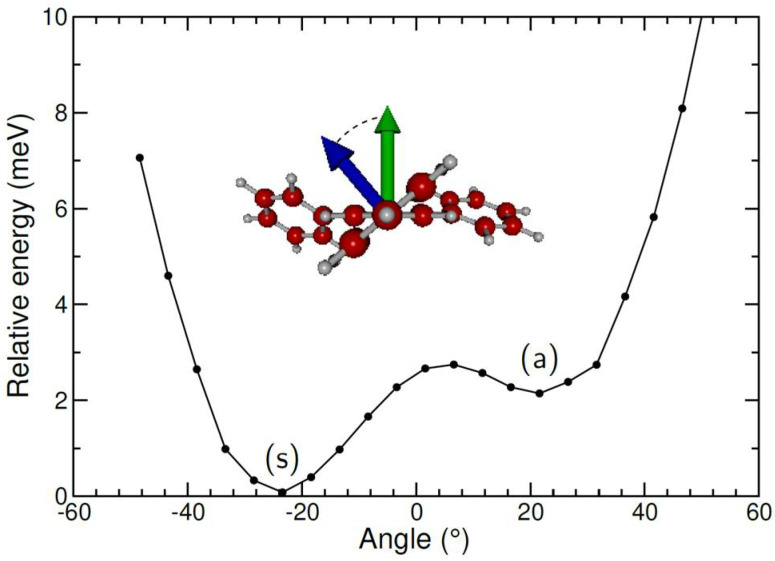
Calculated relative energies for the pathway between the two isomers of TPB^+^, as obtained by rotating the asymmetric phenyl ring around its axis to the central ring.

TPB is a propeller-shaped molecule. In the neutral molecule, calculated with the DFT/B3LYP/6-31G* method, one of the phenyl rings is found to be oriented opposite to the other two rings [39]. For the cation, we find a qualitatively similar “asymmetric” (a) structure. However, a “symmetric” (s) structure, with all three blades oriented similarly, is lower by about 1.7 meV if computed at the quantum chemistry level of DFT/wB97xD/6-31 + G*. An energy pathway was determined using the same method by rotating the phenyl ring around its axis toward the central ring. The resulting energies, shown in Figure 6, further indicate that the energy barrier between these isomers is very small. Furthermore, TPB(s)^+^ becomes higher than TPB(a)^+^ by 3 meV once the zero-point harmonic correction is included. These calculations suggest that the TPB cation could be in a mixture of symmetric and antisymmetric states, or possibly even vibrationally delocalized.

In the limiting case where TPB^+^ assumes a fixed geometry of the (s) or (a) type, global optimization by basin-hopping was performed to identify low-energy structural candidates for 1 to 50 attached He atoms. PIMD simulations were subsequently carried out to determine the corresponding quantum energies. The 2nd derivatives of the computed energies of He*_n_*TPL^+^ are shown in Figure 5c,d for the (s) and (a) isomers, respectively. For both isomers, we predict enhanced stability at *n* = 2, in agreement with the experiment. For TPB(a)^+^, we further predict enhanced stability at *n* = 8, which would coincide with the tentatively proposed feature in the experimental data. For TPB(s)^+^, we predict several additional anomalies (at *n* = 6, 8, 10, 14), clearly at variance with the experimental data.

**Figure 7:**
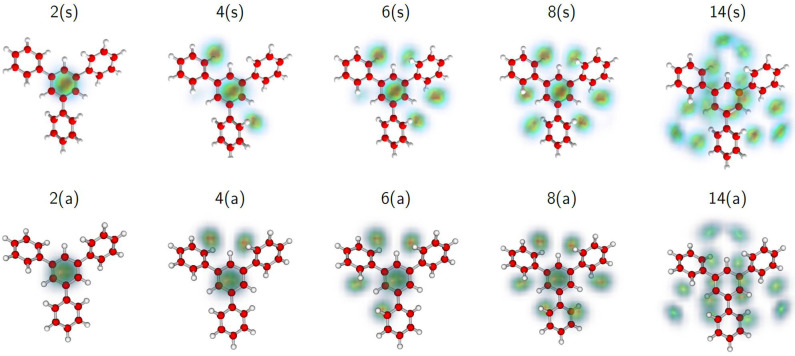
Selected structures of He*_n_*TPB^+^ clusters with *n* = 2, 4, 6, 8, and 14, for isomers (s) and (a). For each size, the helium densities obtained from the PIMD simulations are superimposed on the structure of TPB^+^.

The calculated structure of the adsorbate layer is shown in Figure 7 for *n* = 2, 4, 6, 8, 14, and in Figure S6 for *n* = 10, 12, 16, 18, 30. For small He*_n_*TPB(s)^+^ complexes, the He atoms tend to occupy the hollows of phenyl rings (this is not obvious from Figure 7 because of the tilt of the phenyl rings). The even-numbered complexes prefer a configuration where one half of the adsorbate atoms reside on either side, but the arrangement of the He atoms is not consistent with the approximately 3-fold symmetry of the substrate. For TPB(a)^+^, the adsorption pattern is also rather asymmetric, and He_6_TPB(a)^+^ prefers a 2 + 4 configuration. The particularly stable He_8_TPB(a)^+^ favors the 4 + 4 configuration with each aromatic ring receiving helium atoms on either side.

In our previous work, we investigated helium adsorption on another cationic propeller-shaped molecule, namely hexaphenylbenzene (C_42_H_30,_ HPB) [28]. The orientation of its phenyl groups is known to strongly depend on the environment but, generally, those structures have a 6-fold rotational symmetry for the neutral molecule. Local optimization of HPB^+^ using density-functional theory (DFT) at the wB97xD/6-31 + G* level resulted in a lower symmetry structure (*D*_2_ point group) with two opposite rings at 61° and the other four at 53° only. For this fixed geometry, complexes with *n* = 2, 14, or 28 adsorbed He atoms were calculated to enjoy enhanced stability, in agreement with the experimental data.

The seemingly poorer agreement between experiment and theory for He adsorption on TPB^+^, together with the particularly flat energy pathway predicted by DFT when the phenyl rings rotate around the axes toward the central ring, suggest that the approximation of a fixed geometry for TPB^+^ might be too severe. Moreover, and as for HPB^+^, the orientation of the phenyl rings is also likely to be affected by the mere presence of helium itself, and probably be dependent on the number of adsorbed atoms. A more complete theoretical model accounting for the vibrational delocalization of the TPB cation and its interplay with the surrounding helium atoms, although currently exceeding computational capabilities, would be worth considering further.

## 3. Materials and Methods

### 3.1. Experimental Methods

Cationic He*_n_*TPB^+^ were formed in HNDs and gently extracted as follows: Neutral HNDs were grown by supersonic expansion of helium through a nozzle (diameter 5 μm, temperature 9.4 K, stagnation pressure 27 bar) into ultra-high vacuum. The expanding beam was skimmed and ionized by electrons (energy 66 eV, current 350 μA). The resulting He*_N_^z^*^+^ ions were weakly accelerated into an electrostatic hemispherical deflector set to transmit HNDs with a size-to-charge ratio *N*/*z* ≈ 4.5 × 10^4^.

The charged HNDs passed through a pickup cell into which vapor of TPB (obtained from Sigma Aldrich, stated purity 97%) was introduced from an internal oven kept at or slightly below about 270 °C, and next, in an “evaporation cell” that contained helium at ambient temperature and low, variable pressure *P*_He_. Each collision transfers, on average, 0.05 eV to the HND, about 80 times the evaporation energy of a single helium atom from bulk helium [40]. Multiple collisions lead to partial or complete evaporation of helium from the doped HND. In this process, any multiply charged HNDs will eventually break up into singly charged ions [40,41].

Small He*_n_*TPL^+^ ions were prepared similarly, but the HNDs were formed by a supersonic expansion at a pressure of 22 bar through a nozzle at 9.1 K. The TPL sample (obtained from Sigma Aldrich, stated purity 98%) was vaporized at about 70 °C. (H_2_)*_n_*TPL^+^ was formed by introducing H_2_ at 0.12 mPa into the evaporation cell.

The ions emerging from the helium evaporation cell were guided by a radio-frequency field into the extraction region of a time-of-flight mass spectrometer equipped with a reflectron in V-configuration. The mass resolution was about 5000 (measured at full-width-at-half-maximum) in the mass region of TPB^+^ and TPL^+^. Additional experimental details have been published elsewhere [42].

### 3.2. Computational Methods

He*_n_*TPL^+^, (H_2_)*_n_*TPL^+^, and He*_n_*TPB^+^ clusters were modeled using a combination of classical global optimization techniques and path-integral molecular dynamics simulations, following earlier works [2,28,33]. Briefly, we used a many-body force field to describe the interaction between the adsorbed atoms or molecules and the hydrocarbon ion, consisting of additive repulsion–dispersion interaction between the ligands and the C- or H-atoms of the solvated ion, and a polarizable contribution felt by each ligand resulting from the distribution of partial charges on the hydrocarbon cation. The parameters for the He-cation individual interactions can be found in [33], whereas those for the H_2_-cation, in which H_2_ is treated as a point-like particle, are taken from Ref. [25].

TPL^+^ is planar, and its geometry and partial charges on individual atoms were determined by local optimization using DFT at the wB97xD/6-31 + G* level. The resulting geometry and charges are provided as Supplementary Material (Table S1). The case of bare TPB^+^ turned out to be less straightforward, and local optimizations carried out at the wB97xD/6-31 + G* level now resulted in two distinct, nearly isoenergetic isomers. In the “asymmetric” isomer TPB(a), which is also known as the most stable form in the neutral molecule [39], one blade is oriented oppositely to the two others. In the “symmetric” structure, TPB(s), the three blades of the cation show essentially the same relative orientation and angles relative to the central benzene ring. For both configurations, the partial charges on carbon and hydrogen atoms were determined using single point DFT/wB97xD/6-31 + G* calculations and the standard restrained electrostatic potential (RESP) procedure [43]. The two geometries thus obtained are provided as Supplementary Material (Table S1). All quantum chemical calculations were performed using the Gaussian09 software package [44].

Global optimization by basin-hopping was then performed to identify low-energy structural candidates for 1 to 50 He atoms or H_2_ molecules attached to the hydrocarbon ion, keeping its geometry fixed. For each cluster size, five independent series of 10^5^ local optimizations were carried out, and a fictitious temperature of 10 K was employed to evaluate the Metropolis acceptance probabilities. Nuclear quantum effects were subsequently included by performing path-integral molecular dynamics (PIMD) simulations and evaluating the virial energy of the system, as well as various structural quantities. The PIMD trajectories were carried out at the temperature of 1 K and employed a Trotter discretization number of 128, a time step of 0.5 fs. They were integrated over 1.2 ns, averages being accumulated after 200 ps. Zero-point energy corrections to the static energies of the global minima were also determined in the harmonic approximation but turned out to be too inaccurate to be considered as reliable.

Classical and quantum energies calculated for He*_n_*TPL^+^, (H_2_)*_n_*TPL^+^, He*_n_*TPB(s)^+^, and He*_n_*TPB(a)^+^ are provided as Supplementary Material (Table S2).

## 4. Conclusions

We investigated the adsorption of He or H_2_ on cationic triphenylene (TPL), and of He on cationic triphenylbenzene (TPB). For the planar TPL^+^ the experimental data suggest enhanced stability for complexes containing *n* = 2, 4, or 6 adsorbed He atoms, in reasonable agreement with theory which predicts enhanced stability for *n* = 2 and 6. Theory predicts another particularly stable complex for *n* = 14 but, unfortunately, the experimental data in this size range are compromised by contributions from ions containing two H_2_O molecules.

TPB^+^ is a propeller-shaped molecule; the fluxional character of the orientation of its blades provides an additional challenge to theory. We identify two isomers, one with all three blades oriented similarly, the other with one blade oriented opposite to the other two. For the latter isomer, we computed that *n* = 2 or 8 He atoms would be adsorbed particularly strongly. The value *n* = 2 agrees with experiment, but *n* = 8 agrees only if we postulate that the mass peak assigned to He_9_TPB^+^ has a non-negligible contribution from (H_2_O)_2_TPB^+^. Mass spectra recorded with a deliberately increased water contamination provide credence to this postulate. Alternatively, the very small energy barrier between the two structural isomers of TPB^+^ together with the possible dependence of blade orientation on the presence of ligands already known for hexaphenylbenzene lead to a situation where structures and energies computed for He adsorption on a fixed molecular template are likely to be unrealistic already above two adsorbed helium atoms.

## Data Availability

The data presented in this study are available in the Supplementary Material.

## Supplementary Materials

The following supporting information can be downloaded at: https://www.mdpi.com/article/10.3390/molecules27154937/s1. The file TRI_SI_220712.pdf provides additional experimental and theoretical results in graphical form, plus the calculated geometries of TPL^+^, TPB(s)^+^, and TPB(a)^+^.
