# Supplementary material for: Adsorption of Helium and Hydrogen on Triphenylene and 1,3,5-Triphenylbenzene
Source: Molecules. 2022 Aug 3;27(15):4937. doi: 10.3390/molecules27154937 (PMC9370105; doi:10.3390/molecules27154937)
Supplement: Supplementary file 1 [file molecules-27-04937-s001.zip › molecules-1837369-supplementary.pdf]

Supplementary Material

Adsorption of Helium and Hydrogen on Triphenylene and 1,3,5-Triphenylbenzene

Stefan Bergmeister[1], Siegfried Kollotzek[1], Florent Calvo[2*], Elisabeth Gruber[1], Fabio Zappa[1], Paul Scheier[1], Olof Echt[1,3*]

[1] Institut für Ionenphysik und Angewandte Physik, Universität Innsbruck, A-6020 Innsbruck, Austria
[2] Université Grenoble Alpes, CNRS, LiPhy, F-38000 Grenoble, France
[3] Department of Physics, University of New Hampshire, Durham NH 03824, USA
[*] Corresponding authors:
florent.calvo@univ-grenoble-alpes.fr (Florent Calvo), olof.echt@unh.edu (Olof Echt)

Experimental and theoretical results for triphenylene (TPL, $C_{18}H_{12}$) and 1,3,5-triphenylbenzene (TPB, $C_{24}H_{18}$). The two nearly isoenergetic isomers of TPB^+ are labeled (s) and (a).

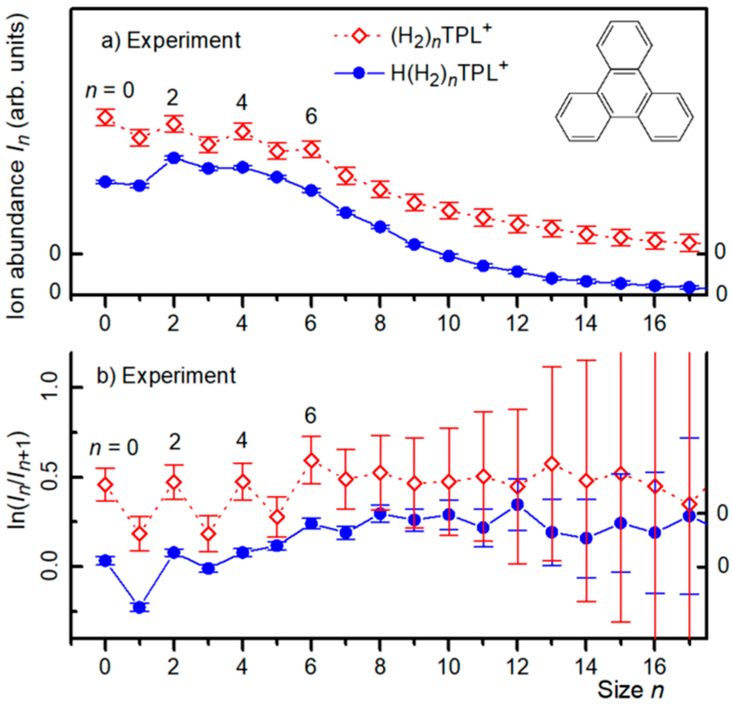

Figure S1. Panel a: Ion abundance I_n of $(H_2)_nTPL^+$ and $H(H_2)_nTPL^+$ versus size n. Panel b: The 1st derivative of the logarithm of I_n. The data sets are stacked for better visibility.

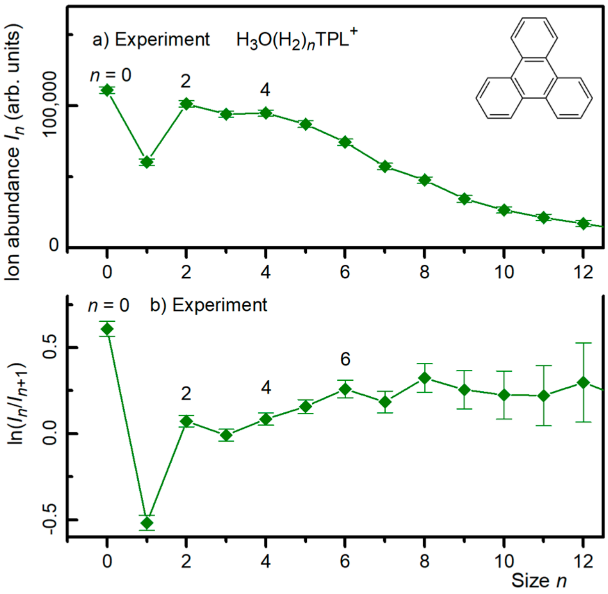

Figure S2. Panel a: Ion abundance I_n of $H_3O(H_2)_nTPL^+$ versus size n. Panel b: The 1st derivative of ln I_n.

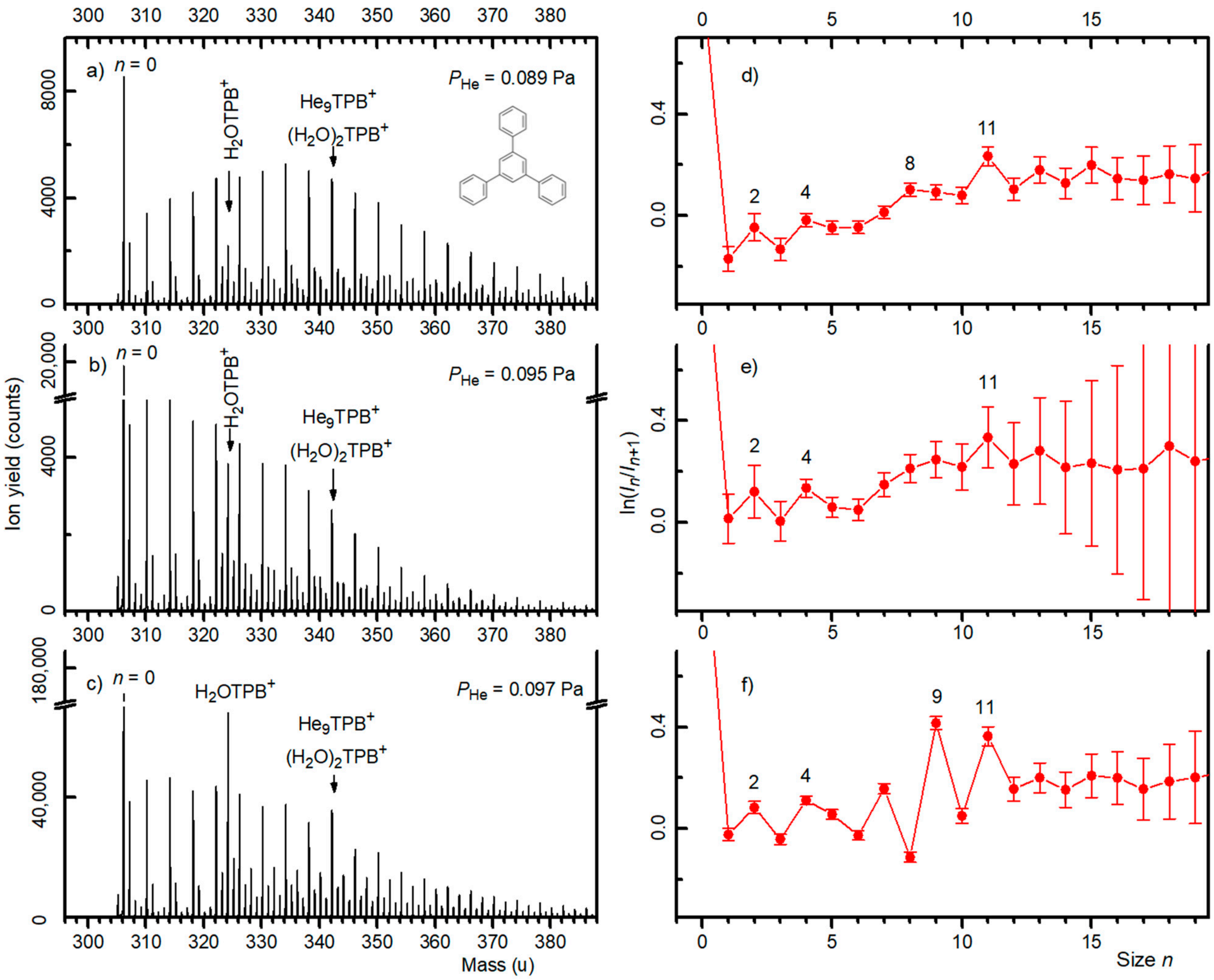

Figure S3. Panels a, b, c: Mass spectra of 1,3,5-triphenylbenzene (TPB, $C_{24}H_{18}$, mass 306.141 u) complexed with helium. The H_2OTPB^+ mass peak shows that the degree of water contamination increases from a to c. Panels d, e, f: The first derivatives of the logarithm of the ion abundance extracted from the respective mass spectra shown on the left. The local maximum at $n = 9$ in panel f is due to the strong contribution of $(H_2O)_2TPB^+$ which cannot be distinguished from the He_9TPB^+ mass peak, but this contribution becomes negligible in panel d as the degree of water contamination decreases.

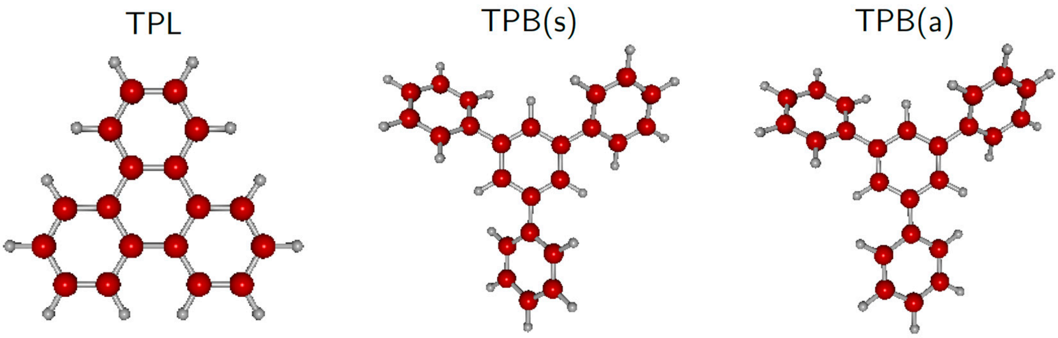

Figure S4. Calculated structures of cationic TPL (planar), and the two non-planar isomers of TPB.

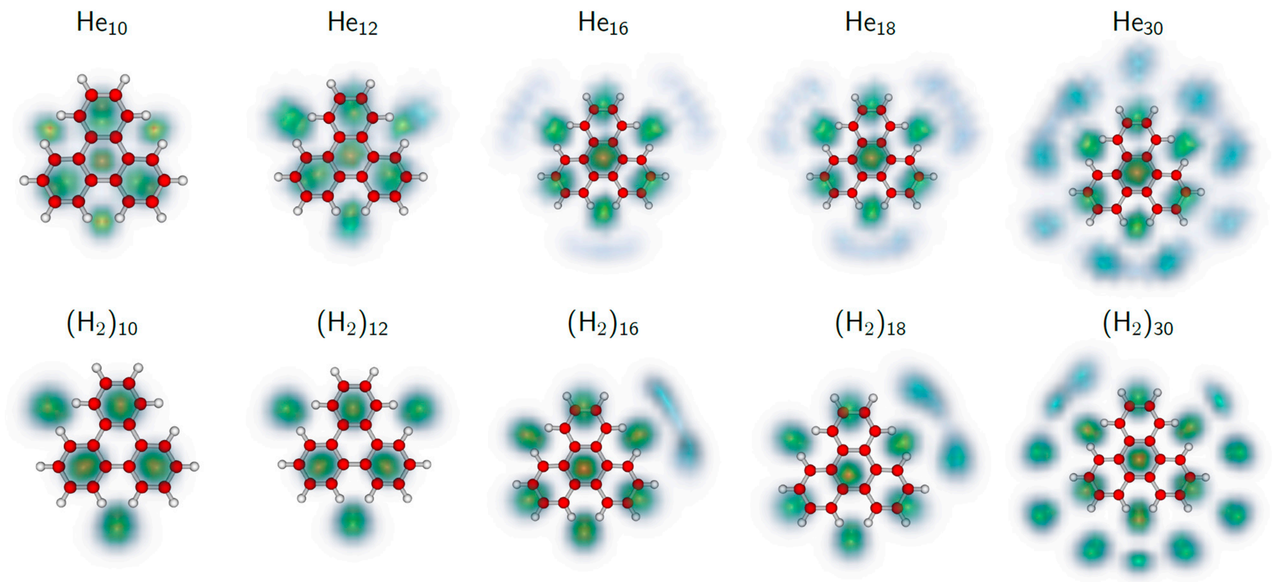

FigureS5. Selected structures of He_nTPL$^+$ and $(H_2)_n$TPL$^+$ clusters with n = 10, 12, 16, 18, and 30. For each size, the He and H_2 densities obtained from the PIMD simulations are superimposed on the structure of TPL$^+$.

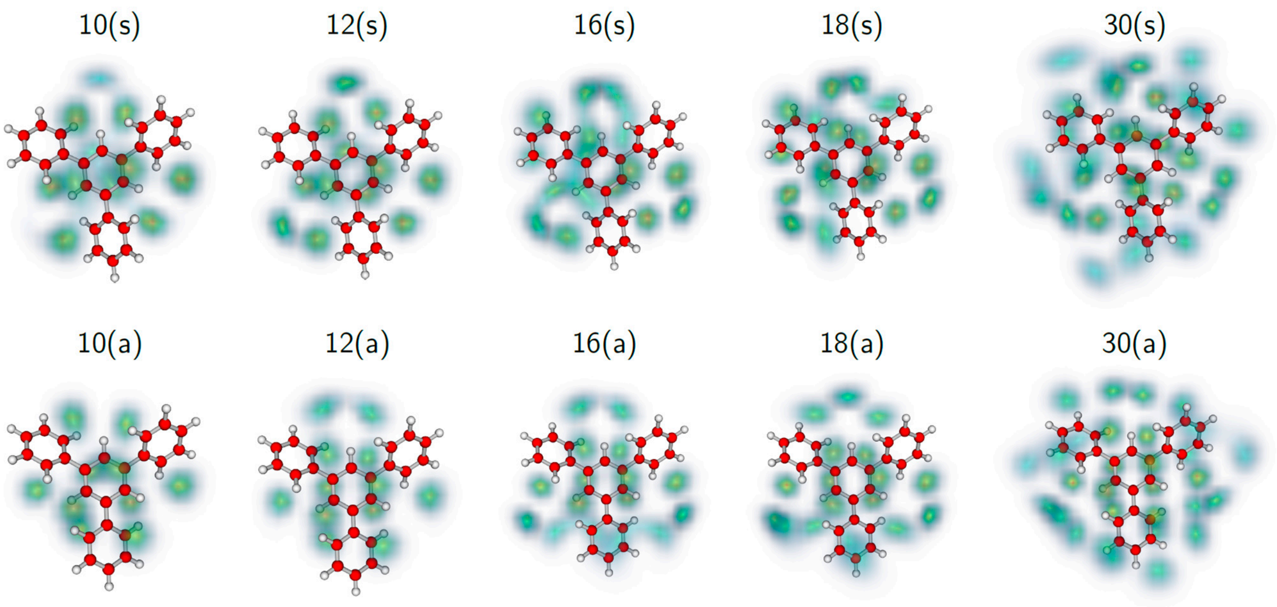

Figure S6. Selected structures of He_nTPB^+ clusters with $n = 10, 12, 16, 18$, and 30, for isomers (s) and (a). For each size, the He densities obtained from the PIMD simulations are superimposed on the structure of TPB^+. 10(s) denotes $He_{10}TPB(s)^+$, etc.

Table S1. Cartesian coordinates (*x*,*y*,*z*) and partial charge (*q*) on each atom of the bare hydrocarbon ion computed with the DFT/wB97xD/6-31+G* method.

Triphenylene TPL^+

	x	*y*	*z*	*q*
C	1.435823	0.005108	0.000000	0.127033
C	0.706972	1.273432	0.000000	-0.007716
C	-0.706972	1.273432	0.000000	-0.007716
C	-1.435823	0.005108	0.000000	0.127033
C	-0.710788	-1.244503	0.000000	0.084518
C	0.710788	-1.244503	0.000000	0.084518
C	2.834163	-0.044914	0.000000	-0.237909
C	1.382609	2.502772	0.000000	-0.091613
C	-1.382609	2.502772	0.000000	-0.091613
C	-2.834163	-0.044914	0.000000	-0.237909
C	-1.456158	-2.471008	0.000000	-0.148277
C	1.456158	-2.471008	0.000000	-0.148277
C	0.694465	3.711162	0.000000	-0.098722
C	-0.694465	3.711162	0.000000	-0.098722
C	-2.821726	-2.477326	0.000000	-0.137357
C	2.821726	-2.477326	0.000000	-0.137357
C	-3.518169	-1.249832	0.000000	0.074069
C	3.518169	-1.249832	0.000000	0.074069
H	-3.367187	-3.414628	0.000000	0.165842
H	3.367187	-3.414628	0.000000	0.165842
H	2.464760	2.541461	0.000000	0.141797
H	-2.464760	2.541461	0.000000	0.141797
H	-4.603876	-1.246213	0.000000	0.138686
H	-0.940704	-3.421492	0.000000	0.163753
H	0.940704	-3.421492	0.000000	0.163753
H	4.603876	-1.246213	0.000000	0.138686
H	3.417729	0.866154	0.000000	0.167297
H	1.246141	4.645373	0.000000	0.158599
H	-1.246141	4.645373	0.000000	0.158599
H	-3.417729	0.866154	0.000000	0.167297

1,3,5-triphenylbenzene $TPB(s)^+$

C	0.595686	1.303911	-0.002677	0.095249
C	-1.413338	-0.091574	0.035623	-0.079394
C	0.848501	-1.128688	-0.031828	-0.034756
C	-0.821488	1.154509	0.054149	-0.076271
C	1.406184	0.132773	-0.057453	-0.106144
C	-0.566155	-1.228065	0.005220	0.119711
C	1.198721	2.615524	-0.003488	0.038678
C	-2.882571	-0.260280	0.022923	0.101480
C	1.679702	-2.351236	-0.019267	0.059581
C	2.498214	2.821598	-0.543632	0.012182
C	-3.683924	0.584288	-0.756958	-0.138572
C	2.860622	-2.394571	0.734119	-0.118425
C	0.512951	3.737098	0.539592	-0.164864
C	-3.490666	-1.264224	0.788427	-0.157242
C	1.301272	-3.478360	-0.761660	-0.144457
C	3.069270	4.077980	-0.549411	-0.033022
C	-5.065685	0.429153	-0.767951	-0.133514

C	3.648653	-3.540066	0.740809	-0.158424
C	1.101932	4.985161	0.553475	-0.138522
C	-4.873272	-1.411276	0.781885	-0.137821
C	2.095743	-4.619469	-0.759485	-0.136842
C	2.378249	5.163476	0.004214	-0.057884
C	-5.662819	-0.566912	0.002576	-0.089108
C	3.269363	-4.652927	-0.007988	-0.089591
H	-1.453883	2.034681	0.081633	0.132387
H	2.485305	0.227819	-0.092400	0.143667
H	-1.019486	-2.215546	0.009276	0.104006
H	3.034156	1.998257	-1.002389	0.103914
H	-3.224093	1.341552	-1.386904	0.127326
H	3.147112	-1.543624	1.346922	0.121114
H	-0.462857	3.610301	0.995162	0.164896
H	-2.888204	-1.913663	1.418672	0.138334
H	0.401002	-3.457621	-1.370947	0.133786
H	4.050956	4.225371	-0.986895	0.115377
H	-5.676179	1.078765	-1.387204	0.152722
H	4.555019	-3.567673	1.337534	0.162166
H	0.577949	5.827492	0.992592	0.181239
H	-5.335612	-2.183159	1.389009	0.157465
H	1.800723	-5.482052	-1.348675	0.157389
H	2.834324	6.148618	0.007235	0.172696
H	-6.741404	-0.688935	-0.007746	0.148545
H	3.884305	-5.547540	-0.002155	0.150941

1,3,5-triphenylbenzene TPB(a)$^+$

C	0.051522	-1.434786	0.010963	-0.250470
C	-1.269371	0.624120	-0.032158	-0.384564
C	1.216357	0.717422	0.051176	-0.384565
C	-1.201021	-0.754391	-0.023621	0.207332
C	1.250270	-0.662321	0.046021	0.207338
C	-0.051465	1.349864	0.009023	0.493514
C	0.106250	-2.877115	0.006044	0.309730
C	-2.558496	1.344722	-0.082626	0.325885
C	2.450656	1.529706	0.090665	0.325879
C	1.267390	-3.563372	-0.444949	-0.205426
C	-3.656340	0.888558	0.659604	-0.280277
C	3.569787	1.151994	-0.663529	-0.280271
C	-0.999965	-3.653887	0.448573	-0.205418
C	-2.700499	2.490070	-0.878230	-0.333282
C	2.518913	2.682899	0.884300	-0.333274
C	1.311217	-4.942791	-0.462914	-0.104874
C	-4.871588	1.561761	0.602980	-0.074061
C	4.734650	1.910062	-0.621433	-0.074064
C	-0.939677	-5.032837	0.449764	-0.104879
C	-3.919223	3.155994	-0.939637	-0.040304
C	3.687918	3.434129	0.931073	-0.040307
C	0.211719	-5.683706	-0.011302	0.029639
C	-5.005896	2.694339	-0.198554	-0.104249
C	4.796482	3.050351	0.177946	-0.104249
H	-2.118926	-1.322900	-0.121129	0.011112
H	2.207482	-1.161606	0.143964	0.011109
H	-0.092322	2.435850	0.008023	-0.086156
H	2.116067	-3.005968	-0.825022	0.200318
H	-3.550797	0.024997	1.311454	0.187325
H	3.518678	0.282233	-1.313770	0.187323
H	-1.887894	-3.166537	0.835356	0.200314

H	-1.868099	2.845880	-1.480278	0.218861
H	1.669643	2.979324	1.495023	0.218858
H	2.195163	-5.452078	-0.831737	0.152385
H	-5.711793	1.208573	1.192536	0.136017
H	5.591254	1.616732	-1.220222	0.136018
H	-1.782771	-5.611931	0.811247	0.152387
H	-4.022244	4.033764	-1.569723	0.128401
H	3.735253	4.317861	1.559575	0.128402
H	0.252691	-6.768588	-0.018354	0.139117
H	-5.955455	3.218416	-0.242795	0.141711
H	5.706527	3.641271	0.210790	0.141712

Table S2. Computed energies versus size n for He_nTPL^+, $(H_2)_nTPL^+$, $(He)_nTPB(s)^+$, and $(He)_nTPB(a)^+$. Classical energies E_c are listed in meV per atom; total quantum energies E_q are listed in hartree.

He_nTPL^+

n	E_c/n (meV)	E_q (har)
0	0	0
1	-20.8614	-0.000547358
2	-20.8678	-0.001095238
3	-19.69607	-0.001514117
4	-19.4648	-0.001936031
5	-19.05378	-0.002348133
6	-19.00149	-0.002771806
7	-18.77591	-0.003121484
8	-18.60826	-0.003481267
9	-18.40244	-0.003809949
10	-18.315	-0.00414966
11	-18.2363	-0.00447712
12	-18.11477	-0.004803059
13	-18.04015	-0.005096007
14	-18.02801	-0.005393754
15	-17.60911	-0.005614286
16	-17.2611	-0.00583146
17	-16.98442	-0.006048502
18	-16.69997	-0.006266072
19	-16.46539	-0.006475996
20	-16.27226	-0.006681098
21	-16.06232	-0.006915579
22	-15.86881	-0.007132551
23	-15.71507	-0.007341397
24	-15.55713	-0.007540491
25	-15.42019	-0.007747757
26	-15.30321	-0.007981299
27	-15.18128	-0.008187127
28	-15.05327	-0.00838678
29	-14.96136	-0.008584397
30	-14.85699	-0.008783918
31	-14.76973	-0.008987686
32	-14.69859	-0.009160633
33	-14.61674	-0.009357977
34	-14.54055	-0.009549094
35	-14.46776	-0.009735707
36	-14.39982	-0.009875835
37	-14.33292	-0.010032477
38	-14.26869	-0.010185185
39	-14.20259	-0.010345545
40	-14.14297	-0.010482762
41	-14.07954	-0.010631475
42	-14.01935	-0.010763064
43	-13.96323	-0.010922564
44	-13.90885	-0.011073517
45	-13.81266	-0.011198673
46	-13.71906	-0.011309316
47	-13.64911	-0.011392151
48	-13.56403	-0.011510629
49	-13.4208	-0.011581119
50	-13.27184	-0.011695085

$(H_2)_n TPL^+$

0	0	0
1	-40.24004	-0.001058501
2	-40.30707	-0.002124176
3	-37.60574	-0.002881821
4	-37.29356	-0.003680061
5	-36.44824	-0.004434703
6	-36.32933	-0.005242985
7	-35.87962	-0.005923754
8	-35.56582	-0.006606863
9	-35.12638	-0.007260163
10	-34.79599	-0.007914293
11	-34.49945	-0.008544275
12	-34.27163	-0.009194343
13	-34.02196	-0.009736447
14	-33.81364	-0.010283942
15	-33.1184	-0.010770767
16	-32.58979	-0.011245971
17	-32.25266	-0.011770618
18	-31.97745	-0.01221868
19	-31.67447	-0.012694915
20	-31.27861	-0.013229421
21	-31.04275	-0.01372147
22	-30.79104	-0.014255316
23	-30.59975	-0.014789229
24	-30.41743	-0.015253224
25	-30.2241	-0.015688048
26	-30.07839	-0.016136315
27	-29.91596	-0.016675612
28	-29.72058	-0.01715919
29	-29.59808	-0.017682648
30	-29.51212	-0.018235005
31	-29.4352	-0.018663137
32	-29.36148	-0.019121178
33	-29.20547	-0.019585011
34	-29.0972	-0.020031517
35	-28.97675	-0.020484282
36	-28.8966	-0.020851754
37	-28.78175	-0.021121692
38	-28.71519	-0.02147632
39	-28.61449	-0.021782685
40	-28.54809	-0.022057304
41	-28.44066	-0.022304596
42	-28.33947	-0.02257148
43	-28.22472	-0.022845181
44	-28.11197	-0.023170903
45	-27.98912	-0.023434787
46	-27.87192	-0.023786507
47	-27.64538	-0.024156059
48	-27.48738	-0.024503527
49	-27.38248	-0.024823783
50	-27.22774	-0.025167675

He$_n$TPB(s)$^+$

0	0	0
1	-20.25989	-0.00054031
2	-20.19602	-0.001079057
3	-19.88823	-0.00155473
4	-19.71468	-0.002023737
5	-19.49996	-0.00247013
6	-19.44152	-0.002918442
7	-19.37681	-0.003319861
8	-19.29294	-0.003804082
9	-19.26078	-0.004209689
10	-19.22748	-0.004615411
11	-19.10319	-0.004963977
12	-18.99952	-0.005322297
13	-18.71795	-0.00565743
14	-18.47312	-0.006002205
15	-18.25814	-0.006275776
16	-18.06871	-0.006554958
17	-17.89327	-0.006843786
18	-17.74694	-0.007098658
19	-17.5849	-0.007353675
20	-17.41127	-0.007585567
21	-17.26454	-0.007819499
22	-17.1199	-0.008067557
23	-16.95913	-0.008305041
24	-16.84283	-0.008523977
25	-16.68633	-0.008749012
26	-16.56762	-0.008965266
27	-16.42997	-0.009190869
28	-16.2856	-0.009399621
29	-16.16124	-0.009614626
30	-16.03207	-0.009826197
31	-15.92527	-0.010016702
32	-15.81474	-0.010182927
33	-15.68559	-0.010365035
34	-15.55049	-0.010545762
35	-15.42257	-0.010731735
36	-15.31755	-0.010935995
37	-15.21804	-0.011093011
38	-15.11973	-0.011238642
39	-15.01864	-0.011421468
40	-14.91153	-0.011600522
41	-14.82867	-0.011776797
42	-14.74237	-0.011963397
43	-14.65755	-0.012145241
44	-14.55821	-0.012294772
45	-14.47977	-0.012431973
46	-14.41018	-0.012575623
47	-14.34153	-0.012726256
48	-14.25415	-0.012873228
49	-14.15988	-0.012989036
50	-14.05928	-0.013116044

He$_n$TPB(a)$^+$

0	0	0
1	-20.28374	-0.000539961
2	-20.26738	-0.001079358
3	-20.0355	-0.001562377
4	-19.88693	-0.002038663
5	-19.79804	-0.002520117
6	-19.72399	-0.002990341
7	-19.58014	-0.003471211
8	-19.49645	-0.003933237
9	-19.39623	-0.004312556
10	-19.32511	-0.004680517
11	-19.18438	-0.005045411
12	-19.06338	-0.005398148
13	-18.82949	-0.005748929
14	-18.64146	-0.006070642
15	-18.45978	-0.006370628
16	-18.32106	-0.006659893
17	-18.16581	-0.006935685
18	-18.05753	-0.00718876
19	-17.8792	-0.007427514
20	-17.70557	-0.007666182
21	-17.49769	-0.007895204
22	-17.33455	-0.008132428
23	-17.16856	-0.008351251
24	-17.03438	-0.008566937
25	-16.8911	-0.008773608
26	-16.75173	-0.008993611
27	-16.6077	-0.009190753
28	-16.47079	-0.009391346
29	-16.31821	-0.009611487
30	-16.18441	-0.009797021
31	-16.05933	-0.010006887
32	-15.94234	-0.010189926
33	-15.81885	-0.010377213
34	-15.70003	-0.010550935
35	-15.56152	-0.010746968
36	-15.43884	-0.010927416
37	-15.31539	-0.011119454
38	-15.206	-0.011302577
39	-15.09393	-0.011461063
40	-14.99226	-0.011622474
41	-14.89381	-0.011803469
42	-14.79963	-0.011948913
43	-14.70622	-0.01212256
44	-14.61358	-0.012277795
45	-14.52531	-0.012435072
46	-14.43951	-0.012592955
47	-14.35404	-0.012733614
48	-14.26712	-0.012879992
49	-14.18234	-0.013044311
50	-14.09481	-0.013185838
